# Association of birthweight with lung function and respiratory diseases: results from the GEIRD study

**DOI:** 10.1093/eurpub/ckac129.561

**Published:** 2022-10-25

**Authors:** I Tocco Tussardi, A Tfaily, L Antonicelli, R Bono, AG Corsico, N Murgia, P Pirina, S Tardivo, DL Jarvis, G Verlato

**Affiliations:** 1 Department of Diagnostics and Public Health, University of Verona, Verona, Italy; 2 Department of Internal Medicine, Ospedali Riuniti di Ancona, Ancona, Italy; 3 Department of Public Health and Pediatrics, University of Torino, Turin, Italy; 4 Department of Internal Medicine, University of Pavia, Pavia, Italy; 5 Section of Occupational Medicine, University of Perugia, Perugia, Italy; 6 Department of Clinical, Surgical Sciences, University of Sassari, Sassari, Italy; 7 National Heart and Lung Institute, Imperial College, London, UK

## Abstract

**Background:**

Early life conditions are associated with lung function and the development of respiratory and non-respiratory illnesses. The relationship with birthweight (BW) is however conflicting. We examined associations of BW with lung function and respiratory diseases within the GEIRD (Gene-Environment Interaction in Respiratory Diseases) study.

**Methods:**

GEIRD is an Italian multi-centre, multi-case control study of people aged 20-84 from the general population conducted from 2008 to 2014. The study included cases of COPD, asthma, allergic rhinitis and controls. Multinomial logistic regression was performed with case/control status (control/COPD/asthma/allergic rhinitis) as response variable, and BW as main determinant adjusting for sex, age and smoking status.

**Results:**

Of 2,287 reporting BW, 6.4 % (n = 147) had low BW (<2500 gr), and this proportion was greater in women than men (7.8% vs. 5.1%; p = 0.006). Lung volumes were significantly lower in individuals with low than normal BW. Median FEV1 was 3.01 L (p25-p75=2.60-3.45 L) versus 3.16 L (2.65-3.86 L) (p = 0.019) and median FVC was 3.68 L (3.19-4.34 L) versus 3.91 (3.34-4.81 L) (p = 0.003). However, FEV1 and FVC were not affected by BW when expressed as percent predicted. Of note, both men and women with low BW were shorter than those with normal BW (mean±SD: 160.2±5.5 vs. 162.6±6.5 cm in women, p = 0.009; 172.4±6.1 vs. 174.8±7.2 cm in men, p < 0.001). FEV1/FVC expressed as absolute ratio or as percent predicted, was not affected by BW. In multinomial analysis, BW was not associated with respiratory diseases in adulthood. However, those with low BW had a higher risk of self-reported hospitalisation for lung disease before age 2 (10.3% vs. 4.1%) and severe respiratory infection before age 5 (16.9% vs. 8.8%) (p = 0.003).

**Conclusions:**

BW was not associated with lung function in adulthood, when controlling for sex and height. Low BW was a risk factor for respiratory diseases in childhood, not in adulthood.

**Key messages:**

• Low birthweight was associated with respiratory diseases in childhood but not in adulthood.

• Although spirometrically-assessed lung volumes were lower in adults with low birthweight, this is likely explained by associations of low birthweight with sex and height.

